# The Effects of Ultrasonic Disintegration as a Function of Waste Activated Sludge Characteristics and Technical Conditions of Conducting the Process—Comprehensive Analysis

**DOI:** 10.3390/ijerph15102311

**Published:** 2018-10-20

**Authors:** Malwina Tytła

**Affiliations:** Institute of Environmental Engineering, Polish Academy of Sciences, 34 M. Skłodowskiej-Curie St., 41-819 Zabrze, Poland; malwina.tytla@ipis.zabrze.pl; Tel.: +48-32-271-6481 (ext. 135)

**Keywords:** waste activated sludge, ultrasonic disintegration, disintegration effects, sludge characteristics, wastewater treatment plant

## Abstract

A comprehensive analysis of the effects obtained in the process of ultrasonic disintegration (UD) of waste activated sludge (WAS), was conducted. Sludge samples were collected periodically from Central Wastewater Treatment Plant (WWTP) in Gliwice (Poland) and disintegrated in the two ultrasonic devices of different construction and technical parameters, i.e., WK-2010 (A) and ultrasonic washer (B). The experiments were performed under a constant energy supply per sludge volume E_V_ = 160 kWh·m^−3^. The direct and technological effects, i.e., after UD and anaerobic digestion (AD) were investigated, respectively. Statistical analysis showed that characteristics and parameters of the WAS, which affects the magnitude of the direct effects create the following sequence: TS (total solids), VS (volatile solids), ΔT (temperature increase) > EPS (extracellular polymeric substances) > SCOD (soluble chemical oxygen demand) > CST (capillary suction time) > N_TOT (_total nitrogen), P_TOT_ (total phosphorus) > pH. Whereas, in the case of technological effects, the above sequence was as follows: TS, VS > CST > N_TOT_, P_TOT_ > pH. Ultrasonic disintegration of WAS prior to AD increased total biogas production (from 13.0% to 19.7%) and reduced the content of TS (from 4.1% to 8.2%) and VS (5.8% to 9.5%) in comparison to the control sample. This confirms the usefulness of ultrasonic disintegration as an effective method of sludge digestion intensification. The obtained results showed that changes in the characteristics of WAS have a significant impact on the magnitude of the effects of ultrasonic disintegration, especially TS, VS, ΔT, EPS, SCOD and CST. Concluding, it can be inferred that the most promising conditions for ultrasonic pretreatment conducted under constant energy supply per sludge volume, are: low power, long sonication time, large surface area of the emitter, and high increase of sludge temperature while conducting the process.

## 1. Introduction

The development of new technologies and growing effectiveness of biological wastewater treatment observed in recent years, can be mainly accounted to the implementation of the European Council Directive [1] concerning urban wastewater treatment, which led to an increase in the amount of sewage sludge production [2,3]. The significance of the abovementioned issue is even more pressing due to the hazards which sludge may pose to the environment and the economy. It is estimated that by the year 2020, the amount of sludge produced in European Union (EU) countries will reach 12,997,000 Mg_TS_, in which 950,000 Mg_TS_ would be in Poland alone [4]. Moreover, the costs associated with sludge treatment and its disposal may constitute as much as 65% of the total operating costs of Wastewater Treatment Plant (WWTP) [5]. Therefore, it is necessary to introduce methods for intensifying anaerobic digestion, which is the most commonly applied process in sludge treatment, that enable sludge stabilization and mass reduction, improve its dewaterability and ensure hygienization [6,7]. Among these methods, we can distinguish disintegration. Nowadays, it is one of the most important environmental issues.

There are different types of disintegration techniques, i.e., mechanical (ultrasonic disintegration, homogenizations), thermal (hydrolysis—low and high temperature), chemical (hydrolysis with oxygen, ozone, sodium hydroxide) and biological (with application of enzymes) [8,9]. Currently, mechanical methods are the most effective and commonly used, especially ultrasonic disintegration, which is based on the cavitation phenomenon [2,10]. Ultrasonic disintegration shortens the hydrolytic phase, i.e., the rate—limiting step of anaerobic digestion (AD), and increases the efficiency of the process [2,6]. Moreover, ultrasonic disintegration (UD) increases the solubility of the organic compounds (by disrupting the sludge floc and cells), leading to a release of intracellular materials available for living organisms, which can be used as a substrate in the subsequent steps of anaerobic digestion [8,11]. It must be emphasized, that ultrasonic disintegration is one of the most secure and environmentally friendly methods of anaerobic sludge digestion intensification, to which mainly waste activated sludge (WAS) is subjected due to the difficulty in its decomposing [8]. The advantages of sludge UD are: lack of byproducts or use of additional reagents and possibility to intervene during the process conducting [11,12]. We can distinguish two types of ultrasonic disintegration effects: direct and technological. The direct effects are observed after ultrasonic pretreatment, whereas the technological during further sludge treatment, i.e., anaerobic digestion [11,13]. The obtained effects are described by appropriate indicators of disintegration, i.a. disintegration degree (DD_COD_) [14], etc. The direct effects are monitored based on the physicochemical changes of sludge characteristics (parameters) before and after pretreatment, i.a. pH [11,15], concentration of soluble chemical oxygen demand (SCOD) [3,16], biogenic substances [17,18] and extracellular polymeric substances [9,19] in sludge supernatant, capillary suction time (CST) [7,20] or microscopy examination of a flocs disruption [5,21]. In the case of anaerobic digestion the expected effects are: increase in biogas production, total solids (TS) and volatile solids (VS) reduction, as well as dewatering ability improvement [10,13]. The effects of sludge pretreatment are also influence by operating parameters of the process conducting, i.a. technical construction of ultrasonic device, frequency (f), the amount of energy supplied per sludge volume (E_V_) or total solids content (E_S_), ultrasonic density (U_D_) and intensity (U_I_), sonication time (t) and process temperature (T) [18,22,23].

Therefore, taking into account the number of factors, which may have an impact on a magnitude of disintegration effects, a comprehensive analysis and selection of the most important sludge characteristics (parameters) and technical conditions of the process conducting are necessary. This is particularly important due to the fact that it is still unknown why in the similar conditions of sludge sonication, different effects are obtained. However, it is important to emphasize that there are no two identical sludges—their characteristics change over time. Therefore, it is important to carry out the ultrasonic disintegration in a cyclical manner, i.e., at an appropriate time intervals, which will allow observing changes in sludge characteristics and indicate the main parameters, that have the greatest impact on the obtained effects.

The aims of this study were: (a) to evaluate direct and technological effects based on the values of selected indicators and optical microscopy examination; (b) to determine sludge characteristics (parameters) and technical conditions of the process conducting, having the greatest impact on the direct and technological effects; (c) to conduct a comprehensive analysis of the disintegration effects, with including periodically changes of WAS characteristics and technical conditions of conducting the process, using selected statistical tests.

## 2. Materials and Methods

### 2.1. Sludge Collection and Analysis

The WAS samples were collected once a month over a 7-months period of time, starting from July 2013 up to January 2014. Sludge samples after mechanical thickening were collected from the advanced biological Central Wastewater Treatment Plant in Gliwice (Poland, Central Europe). The digested sludge (inoculum) was taken from a full scale anaerobic digester (mesophilically operated) in the same WWTP and used only in order to conduct anaerobic digestion. The collected sludge samples were stored in polypropylene tubes at 4 °C before further analysis. The operational parameters of the WWTP are reported in Table 1.

In order to determine the changes of sludge samples characteristics—before and after UD/AD, selected parameters were considered, i.e., pH, SCOD, N_TOT_ (total nitrogen), P_TOT_ (total phosphorus), proteins and carbohydrates concentrations, CST, flocs disruption (microscopic examination; at 100× magnification), TS and VS content, as well as volume and composition of the evolved biogas. Prior and after UD or AD, sludge samples were centrifuged for 30 min at 20,000 rpm (18 °C) and subjected to vacuum filtration throughout membrane filters (0.45 μm; Chemland, Gyeonggi-do, Korea) [24]. Sludge prepared in the following manner was measured for: SCOD, N_TOT_ and P_TOT_ concentrations. In order to determine the concentration of extracellular polymeric substances the thermally extraction was conducted. At the beginning, WAS sample was centrifuged at 2000× g for 20 min. The residual sludge pellet was resuspended in the distilled water right to the original volume and placed into the water bath (at 80 °C) for 1 h. After incubation sludge was separated from extract by centrifugation at 2000× g and 4500× g over 20 min each time, respectively [25,26]. In the obtained extracts the concentration of proteins and carbohydrates, were measured. The albumin bovine (Acros Organics) and glucose (POCH), were used as a standard solutions. Moreover, the measurements of: pH, TS, VS, CST and sludge temperature increase (ΔT), as well as microscopic examination, were determined only in the sludge samples. Whereas, the biogas volume and composition (CH_4_, CO_2_, H_2_S) were measured during sludge anaerobic digestion. The list of methods/devices used for sludge samples analysis, before and after UD and/or AD processes (including type of the obtained effects) are shown in Table 2.

### 2.2. Experimental Design and Operating Conditions

#### 2.2.1. Ultrasonic Disintegration

Two different experimental ultrasonic devices were applied for WAS ultrasonic disintegration. The first device (A) consists of disintegrator of high power disintegrator WK-2010, equipped with “sandwich” head and “lens” emitter (designed and manufactured by SemiInstruments, Zabrze, Poland). This device is also equipped with a gauge allowing to read the frequency, as wells as with a steel chamber where the UD of sludge samples takes place. The second device (B) consists of ultrasonic washer which is a rectangular chamber, equipped with a single “flat” emitter of “sandwich” type placed in the bottom of the chamber (designed and manufactured by ZUT Intersonic S.C., Olsztyn, Poland). The technical characteristics and operating conditions of the experimental devices are shown in Table 3.

The WAS samples pretreatment were performed under a constant energy supply (over time) per sludge volume, i.e., E_V_ = 160 kWh·m^−3^ (E_S_—in the range of 10,868–23,226 kJ·kg^−1^ TS; app. 15,111 kJ·kg^−1^ TS). Sludge samples were disintegrated for: 267 s (WK-2010) and 1920 s (ultrasonic washer). The sample volume was constant and equal V = 0.3 L. The ultrasonic density (U_D_) for each of experimental device was as follows: 2.2 W·cm^−3^ (WK-2010) and 0.3 W·cm^−3^ (ultrasonic washer). Whereas, the ultrasonic intensity (U_I_) was: 2.6 W·cm^−2^ (WK-2010) and 1.9 W·cm^−2^, respectively. The above conditions were the most favorable ones and were determined in the course of previously carried out studies. The variable parameter during sludge UD was the amount of specific energy (E_S_), which depends on TS content. The values characterizing the ultrasound field and the amount of energy supplied to the process were calculated by the following Equations (1)–(4):

(1) $$E_{V}=\frac{P\times t}{V}$$

(2) $$E_{S}=\frac{P\times t}{V\times \mathrm{TS}}$$

(3) $$U_{D}=\frac{P}{V}$$

(4) $$U_{I}=\frac{P}{A_{E}}$$

where: E_V_—energy supplied per sludge volume (kWh·m^−3^) [16]; E_S_—specific energy (kJ·kg_TS_^−1^) [17]; U_D_—ultrasonic density (W·cm^−3^) [36]; U_I_—ultrasonic intensity (W·cm^−2^) [16]; P—power of the ultrasonic generator (W; kW); V—volume of a sludge sample (m^3^·cm^3^); t—sonication time (s); TS—total solids; (g·L^−1^); A_E_—surface area of the disintegration (cm^2^).

#### 2.2.2. Anaerobic Digestion

The anaerobic digestion was conducted in the installation consisting of the anaerobic glass digesters with a working volume of 0.5 L, water bath—in order to maintain a constant temperature, columns to collect and measure the volume of evolved biogas and MULTITEC 540 gauge (Sewerin, Warszawa, Poland)—applied for composition analysis. AD was conducted under mesophilic conditions (37 °C ± 0.5 °C) over 20 days, each time. The examined sample was a mixture of: digested sludge (30%) collected from the fermentation chamber of the Central WWTP in Gliwice and ultrasonically pretreated one (70%) from the same WWTP. Control sample constituted mixture of untreated and digested sludge. During the AD process each digester was shaken manually three times per day to prevent the sludge from settling. The process was conducted once a month (for a period of 7 months). The research position was made by Wytwórnia Przyrządów Laboratoryjnych (WPL) Gliwice, according to DIN 38414-8:1985 [37].

### 2.3. Direct and Technological Effects

In order to determine the direct and technological effects of sludge UD process, selected indicators were used (defined in Section 2.3.1). The assessment of the direct effects included changes of: pH value, concentration of SCOD, P_TOT_, N_TOT_, proteins and carbohydrates, as well as CST measurement and sludge flocs optical microscopy analysis. Whereas, the technological effects observed after completion of sludge AD included the same parameters as for the direct once (except SCOD, extracellular polymeric substances (EPS) and microscopic analysis). Moreover, process of AD was also controlled by the volume and composition of the evolved biogas, as well as by measuring the rate of TS and VS reduction.

#### 2.3.1. Indicators of the Direct and Technological Effects

In order to evaluate the direct and technological effects of WAS ultrasonic disintegration, a comprehensive analysis of the obtained results was conducted. The disintegration degree (DD_COD_) was determined using indicator proposed by Müller [14] (Equation (4). Whereas, the magnitude of the obtained effects was assessed using author’s indicators (ID_i_, IT_i_, IT_d_) based on the ratio of the concentration or value of specific compound resulting from changes in the sludge characteristics, as a result of its pretreatment [13] (Equations (5) and (7)):

(5) $${\mathrm{DD}}_{\mathrm{COD}}=\frac{{\mathrm{SCOD}}_{\mathrm{UD}}-{\mathrm{SCOD}}_{0}}{{\mathrm{SCOD}}_{\mathrm{NaOH}}-{\mathrm{SCOD}}_{0}}\times 100$$

where: DD_COD_—disintegration degree of (%), SCOD_0_ and SCOD_UD_—supernatant COD of the original and disintegrated sample (mg·L^−1^), SCOD_NaOH_—the maximum COD_NaOH_ obtained by alkaline hydrolysis (0.5 M NaOH, ratio of 1:1 for 22 hours at 20 °C) (mg·L^−1^).

(6) $$\mathrm{IDi};\mathrm{ITi}=\frac{C_{\mathrm{UD}}}{C_{\mathrm{NUD}}}$$

(7) $$\mathrm{IDd};\mathrm{ITd}=\frac{C_{\mathrm{NUD}}}{C_{\mathrm{UD}}}$$

where: IDi—direct effects indicator relating to the increase of concentration or value of specific compound in sludge/supernatant, in the process of UD; ITi—technological effects indicator relating to the increase of concentration or value of specific compound in sludge/supernatant, in the process of UD, observed after AD; IDd—direct effects indicator relating to the decrease of concentration or value of specific compound in sludge/supernatant, in the process of UD; ITd—technological effects indicator relating to the decrease of concentration or value of specific compound in sludge/supernatant, in the process of UD, observed after AD; C_NUD_ and C_UD_—concentration or value of a specific compound in the sludge/supernatant of non—disintegrated and disintegrated sludge, respectively (mg·L^−1^), (s).

### 2.4. Statistical Analysis

In order to evaluate the obtained results, a comprehensive statistical analysis was conducted. The calculations were performed with Statistica 12.0 (StatSoft) and Excel 2013 (Microsoft Office Standard). To check the differences in the mean concentrations of specific compounds between related groups of variables (sludge characteristics before and after UD and AD processes), *T*-Test was used. The occurrence of a linear correlation between analyzed variables was evaluated by Pearson’s correlation coefficient (r). To determine whether any of the differences between the means are statistically significant, one-way analysis of variance (ANOVA) was used. Tests were carried out with a confidence level of 95%.

## 3. Results and Discussion

### 3.1. Direct Effects

The characteristics of WAS before and after ultrasonic pretreatment are shown in Table 4. The values of parameters analyzed in WAS (collected periodically for 7 months), were in the range of: 6.9–7.2 (pH); 24.8–53.2 g·L^−1^ (TS); 24.8–53.2 g·L^−1^ (VS); 46.6–82.7 mg·L^−1^ (SCOD); 8.2–12.4 mg·L^−1^ (N_TOT_), 16.2–37.7 mg·L^−1^ (P_TOT_); 624.6–1228.2 mg·L^−1^ (proteins); 241.7–821.7 mg·L^−1^ (carbohydrates) and 7–13 s (CST). Among all examined parameters, the pH was characterized by the lowest variability (CV = 2.0%), whilst the concentration of carbohydrates by the highest one (CV = 35.6%).

**Figure A1:**
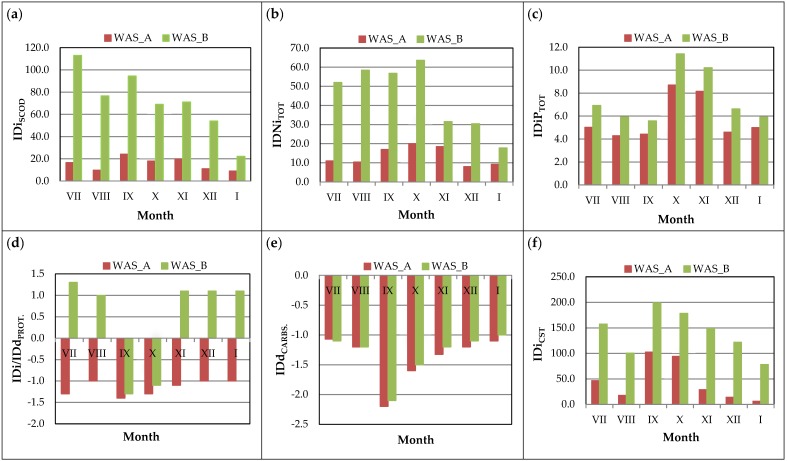
Values of indicators of the direct effects of WAS ultrasonic disintegration: (**a**) IDi_SCOD_; (**b**) IDi_NTOT_; (**c**) IDi_PTOT_; (**d**) Idi/IDd_PROT_; (**e**) IDd_CARBS_; (**f**) IDi_CST_. WAS_A—waste activated sludge after pretreatment in the WK-2010; WAS_B—waste activated sludge after pretreatment in the ultrasonic washer.

As a result of WAS pretreatment, the pH value slightly decreased and was in the range from 6.7 to 7.2 and 6.5 to 7.1, for sludge disintegrated in the WK-2010 (WAS_A) and ultrasonic washer (WAS_B), respectively. The highest variability was observed for sludge pretreated in the ultrasonic washer (CV = 2.7%). The decrease of pH was also confirmed by IDd_pH_, which values were lower than 1.0 (for this reason, it was not included in Figure A1). The above observations were in good agreement with author’s previous research [13]. The influence of ultrasonic pretreatment on decreasing of pH value, was also confirmed by other scientist [38], who claimed that it is probably posed by formation of acidic compound resulted from flocs disintegration. Whereas, other researches indicated that ultrasonic pretreatment did not change the pH of sludge [39]. It was also suggested that for effective sludge ultrasonic disintegration, the value of pH must be adjusted to a suitable level [15,40].

**Figure 1:**
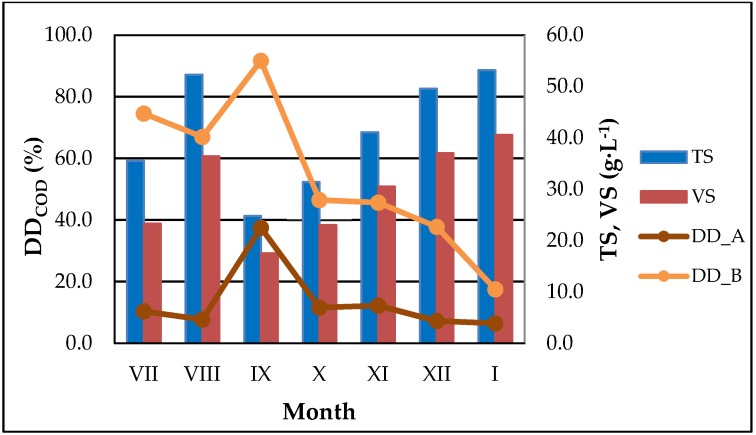
Evolution of disintegration degree (DD_COD_) vs total solids (TS) and volatile solids (VS) content of waste activated sludge (WAS).

**Figure 2:**
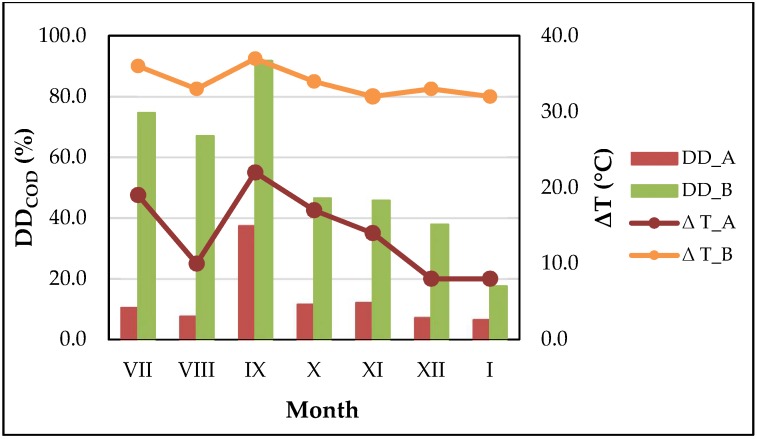
Evolution of disintegration degree (DD_COD_) vs temperature increase (ΔT) of waste activated sludge (WAS).

The concentrations of SCOD in the supernatant after sludge pretreatment, were in the range of 741.0–1827.0 mg·L^−1^ (WAS_A) and 1842.0–6935.0 mg·L^−1^ (WAS_B). Higher variability of examined parameter was observed for sludge undergone disintegration in the ultrasonic washer (CV = 39.2%). The biggest increase of SCOD concentration in the sludge supernatant was noted in July and September, when the TS content was in the range of 24.8–35.5 g·L^−1^. The above observations were confirmed by DD_COD_ and IDi_SCOD_, which maximum values amounted: 37.5 and 91.7% (Figure 1), as well as 24.1; 113.0 (Figure A1) for WAS pretreated in WK-2010 and ultrasonic washer, respectively. The influence of TS content on SCOD release was also confirmed by the other researchers, who claimed that its optimal value should be in the range of 2.0–3.2% [11,41]. However, there have been many studies reporting an increase of SCOD concentration in the supernatant after sludge ultrasonic pretreatment. For example, it was indicated that after 40 min of sludge pretreatment conducted at E_S_ = 9690 kJ·kg^–1^_TS_, DD_COD_ reached 57.9% [18]. Moreover, other researchers indicated that the SCOD solubilisation and disintegration degree may depend on the amount of energy input, sonication time or frequency of ultrasonic wave [3,10,16]. However, there is one more important factor, which affects the release of SCOD into supernatant, i.e., the increase of sludge temperature. The conducted experiment showed, that depending on the type of device used for sludge sonication, the increase of sludge temperature ranged from 8 to 22 °C (WK-2010) and 32 to 37 °C (ultrasonic washer). Furthermore, the highest values of ΔT were observed at the lowest TS content and amounted: 22 and 37 °C, respectively (Figure 2). The above observations were confirmed by other researcher, who indicated that at a constant energy input (E_V_ = 100 kWh·m^−3^), higher increase of sludge temperature was obtained at lower TS content, i.e., from 27.2 to 38.2 °C at TS = 2.0% and 18.0 to 29.0 °C at TS = 4.2% [23]. Moreover, the results of another research revealed, that the increase of sludge temperature may increase linearly with increasing value of specific energy input to the process, i.e., temperature of sludge increased from 22 °C in original sample to 72 °C for disintegrated one, at a maximum E_S_ = 15,880 kJ·kg^−1^ TS [18]. It is important to note that, the increase of WAS temperature during ultrasonic pretreatment generally has a positive influence on sludge solubilisation, but to avoid of any undesirable effects relating, e.g., with recycling back of leachate (generated after sludge pretreatment) to the biological process, it must be undergo testing and further controlled.

This study revealed that ultrasonic pretreatment of WAS increased the concentration of biogenic substances in the sludge supernatant. The concentration of total nitrogen ranged from 99.5 to 196.0 mg·L^−1^ (WAS_A) and 210.0 to 719.0 mg·L^−1^ (WAS_B), while for the total phosphorus from 91.5 to 187.0 mg·L^−1^ and 126.0 to 223.0 mg·L^−1^, respectively. The concentration of N_TOT_ was characterized by higher variability during the experiment duration. The value of CV for N_TOT_ and P_TOT_ were in the range of 23.8–37.5% and 18.7–22.5%, respectively. Almost for all examined sludge samples, the highest values of IDi_NTOT_ and IDI_PTOT_ were obtained in October, i.e., 20.0 (WAS_A), 63.6 (WAS_B) and 8.7 (WAS_A); 11.4 (WAS_B) respectively (Figure A1), while the lowest in January. The above effects were probably additionally strengthened by the increase of temperature during sludge ultrasonic pretreatment. The obtained results are in good agreement with author’s earlier research [13]. Moreover, in the scientific papers little attention is given to the release of biogenic substances in the supernatant during the ultrasonic pretreatment of sludge. However, some of the conducted research indicated that increase of specific energy increased the concentration of total nitrogen and phosphorus by 716% and 207.5%, respectively (at E_S_ = 9690 kJ·kg^−1^ TS) [18]. It is important to note that most part of nitrogen and phosphorus in the sludge supernatant existed in the form of organic products. Thus, the increase of biogenic substances concentration in the supernatant after sludge pretreatment may be associated with the concentration of SCOD or extracellular polymeric substances [17,18]. The above finding was confirmed in this work.

As a result of ultrasonic disintegration of WAS, for most of the analyzed sludge samples the decrease in proteins concentration were observed. The only exception was shown for the sludge samples pretreated in ultrasonic washer, where the increase of proteins content was observed (except of September and November, when TS content was the lowest and ΔT almost the highest). The proteins concentration in the supernatant fluctuated in the range from: 465.3 to 1213.5 mg·L^−1^ (WAS_A) and 618.5 to 1310.9 mg·L^−1^ (WAS_B). The above results were confirmed by the values of IDi_PROT._ and IDd_PROT_., which ranged from: −1.0 to −1.4 (WAS_A) and −1.3 to 1.3 (WAS_B) (Figure A1). Moreover, similar observations were made for carbohydrates, where their concentration in the supernatant was in the range of: 108.3–754.2 mg·L^−1^ (WAS_A) and 115.0–790.0 mg·L^−1^ (WAS_B). The values of IDd_CARBS_ amounted: −1.1 to −2.2 (WAS_A) and −1.0 to −2.1 (WAS_B) (Figure A1). The highest variability of above extracellular polymeric substances was observed for sludge samples disintegrated in the WK-2010, i.e., CV = 35.1% and CV = 47.5%, for proteins and carbohydrates, respectively. Moreover, sum of EPS, during whole time of experiments duration were decreased. The obtained results were in the good agreement with those obtained by other researchers. For example, it was claimed that the increase of EPS concentration in the supernatant after sludge ultrasonic pretreatment (ES in the range of 0.1–50 kJ·kg^−1^ TS) increased the concentration of above substances initially, but with increasing energy supplied to the process their content decreased [19]. Whereas, other researchers indicated that ultrasonic pretreatment of WAS, conducted at high level of specific energy (up to 26,000 kJ·kg^−1^ TS) increased the concentration of EPS in the sludge supernatant [17]. However, it must be emphasized, that in the above mentioned study, sludge temperature raised only by 4 °C. It is important to note that proteins and carbohydrates constitute one of the most important components of EPS (they are part of sludge flocs). Thus, its presence in sludge play a significant role in regulating sludge dehydration ability [42], which was confirmed in this work. For example, some researchers indicated that sludge dewaterability may initially increase with the increase of EPS and then decreased when EPS content exceeded a certain threshold [43].

Ultrasonic pretreatment of WAS caused deterioration of the sludge dewaterability expressed by the increase of CST value, which ranged from 147 to 627 (A) and 1042 to 1396 (B). The highest values of CST were noted in September, while the lowest in January, when the TS content was 24.8 g·L^−1^ and 53.2 g·L^−1^, respectively. The values of IDi_CST_ were in the range of 6.2–102.6 (WAS_A) and 78.2–199.4 (WAS_B) (Figure A1). Similar observations were made by other researcher, who claimed, that at constant energy input to the process of sludge ultrasonic pretreatment, higher values of CST were obtained at lower TS content [11]. Other scientists indicated that CST may increase with an increase of specific energy input to the process [7]. Moreover, generally it was revealed that CST = 20 s is regarded as representative for a good dewatered sludge [44]. In this study, deterioration of sludge dewaterability was probably caused by the fragmentation of solid fraction and weakens the internal structure, which resulted in the increase of sludge surface characterized with very small particles. Deterioration of the sludge dehydration could also be associated with the release of EPS during sludge pretreatment [42].

**Figure 3:**
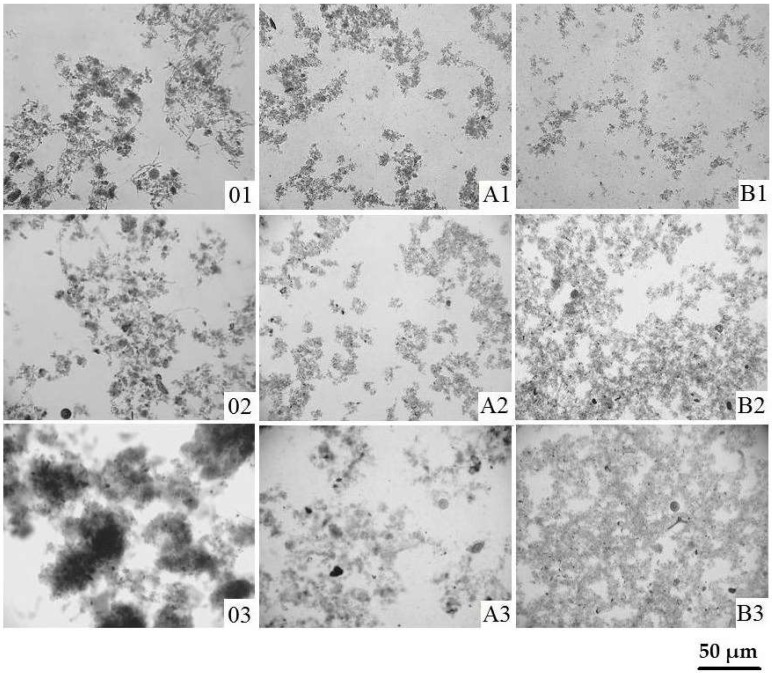
Photomicrographs of the: non-disintegrated (**01**–**03**) and disintegrated WAS (**A**–WK-2010; **B**–ultrasonic washer) at different total solids (TS) content: 24.8; 35.5 and 53.2 g·L^−1^, respectively (100× magnification).

In order to compare the changes in flocs structure, occurring as a result of WAS pretreatment, three samples were selected and subjected to microscopic examination. They differed in TS content. The first one (S1) was characterized by a lowest TS (24.8 g·L^−1^); second one (S2) by mean (35.5 g·L^−1^) and third one by a highest TS content (53.2 g·L^−1^), among of all samples analyzed during seven months of the experiment conducting. The collection of above samples was conducted in: September, July and January, respectively. The results were presented depending on the type of disintegrator used for sludge pretreatment, i.e., WK-2010 (A1–A3) and ultrasonic washer (B1–B3) (Figure 3). The optical microscopy analysis indicated significant differences in the flocs structure before and after sludge pretreatment. The most visible changes were observed for WAS disintegrated in the ultrasonic washer. A strong breakdown and dispersion of the flocs were observed. The experiment showed, that both sludge characteristics and technical conditions of process conducting, have a considerable impact on the sludge structure. The above observations were confirmed in author’s previous studies [13,24]. Moreover, the usefulness of microscopic analysis in assessing the direct effects of sludge ultrasonic disintegration, has also been demonstrated by other researchers [5,17].

### 3.2. Technological Effects

The characteristics of inoculum, as well as samples containing untreated or disintegrated WAS (mixed with inoculum), before (S_0; S_A; S_B, respectively) and after the process of AD (S_0*; S_A*; S_B*, respectively) are shown in Table 5. 

It was indicated that mixtures containing WAS after ultrasonic pretreatment (S_A; S_B), were characterized by higher concentrations and values of examined parameters, compared to the control sample (S_0). The highest variability in the mixtures before AD was expressed by P_TOT_ concentration and CST values. Similar relations were observed in with reference to mixtures after AD process. For both samples, before and after AD, the lowest variability was observed for pH value. 

**Figure A2:**
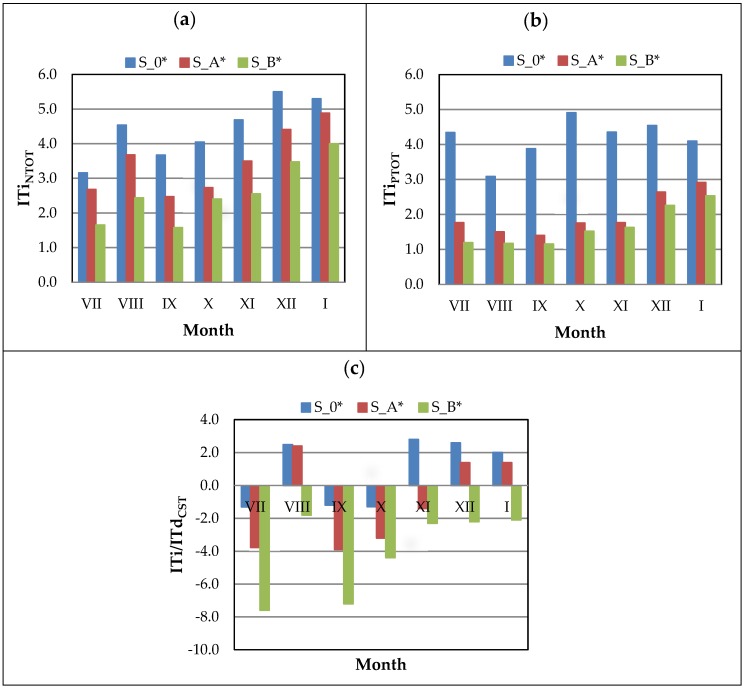
Values of indicators of the technological effects of WAS ultrasonic disintegration: (**a**) ITi_NTOT_; (**b**) ITi_PTOT_; (**c**) ITi/ITd_CST_. S_0*—sample after AD containing inoculum and original sludge; S_A*—sample after AD containing inoculum and WAS_A; S_B*—sample after AD containing inoculum and WAS_B.

The conducted experiment indicated, that after AD process, following changes in the examined mixtures were observed: the increase of pH, N_TOT_ and P_TOT_ concentration, as well as the reduction of TS and VS content and increase of biogas production. While, CST values were generally decrease. The above observations were confirmed by ITi and ITd (Figure A2). Moreover, due to the low values of Iti_pH_ (<1.0), this parameter was not included in Figure A2.

As a result of ultrasonic disintegration of WAS, in the mixture derived from anaerobic digestion process a high increase of N_TOT_ and P_TOT_ concentration, were observed. The values of ITi_NTOT_ and ITi_PTOT_ ranged: 3.2–5.5; 2.5–4.9; 1.6–4.0 and 3.1–4.9; 1.4–2.9; 1.2–2.5, for S_0*; S_A* and S_B*, respectively (Figure A2). The lowest increase of above mentioned parameters was observed for the mixtures containing sludge after pretreatment in the ultrasonic washer, for which the temperature increase was the highest. It was also indicated, that the increase of biogenic substances in sludge supernatant after AD process was higher in the control sample. The above observations were confirmed by author’s previous work, in which ITi_NTOT_ values for the reference samples were in the range of 3.2–5.7 and for mixtures containing disintegrated sludge equaled 2.4–5.5. While in respect to ITiP_TOT_, those values were from 3.5 to 4.9 and 1.5 to 2.8, respectively [13]. Moreover, the increase of biogenic substances in the supernatant after anaerobic digestion of WAS, was associated with the increase of TS and VS content, as well as the decrease of CST value in the samples before the process, which resulted from characteristics of sludge undergone ultrasonic disintegration. Furthermore, the changes in the concentration of total nitrogen in the supernatant after and before sludge AD, were correlated with the increase of biogas production. Whereas, in the case of the total phosphorus, changes in the concentration of this compound, were related with the amount of evolved biogas. The increase in concentration of biogenic substances in the supernatant after sludge AD, was also observed by other researchers [45]. Moreover, these findings can be positive with respect to necessity of recycling the leachate generated after dehydration of digested sludge into the technological line of WWTP.

**Figure 4:**
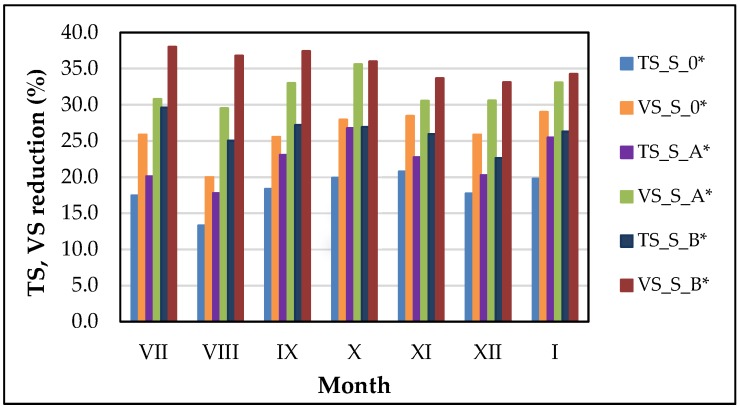
Total solids (TS) and volatile solids (VS) reduction after anaerobic digestion (AD) process.

The reduction of TS and VS content in the samples containing sludge after UD was in the ranged of: 17.8–26.8%; 22.6–29.6% and 29.5–35.6%; 33.1–38.0%, for S_A*; S_B*, respectively. It was showed that the obtained TS and VS reduction was higher in comparison to the control sample by: 4.1; 8.0% and 5.8; 9.5%, for S_A*; S_B*; respectively (Figure 4). The highest decrease of TS and VS content was observed for samples containing WAS after pretreatment in the ultrasonic washer, compared to the control sample. Moreover, the reduction of TS and VS content increased the biogas production. The above results were in good agreement with other researchers, who stated, that ultrasonic pretreatment of sludge, increased VS reduction and biogas production by: 19% and 26%, respectively [46]. Whereas, the results of other investigation, indicated that ultrasonic pretreatment of sludge prior anaerobic digestion, ensured: 12% increase of TS content, compared to the control sample [21].

The changes of CST values in the sludge mixtures after anaerobic digestion revealed, that the best effects of sludge dehydration were obtained for samples containing WAS, after pretreatment in the ultrasonic washer. The values of ITi_CST_ and ITd_CST_ were in the range of: −1.3 to 2.8; −3.9 to 2.4; −1.8 to −7.6, for S_0*; S_A* and S_B*, respectively (Figure A2). The best effects were obtained in the first, third and fourth month of experiments procurement. The obtained results are probably dependent on the characteristic of sludge samples after ultrasonic pretreatment, i.e., the worse susceptibility of sludge to dewatering (larger surface area of the flocks), the greater degree of sludge defragmentation, which favors the course of the anaerobic digestion process [8]. The above observations were in a good agreement with those obtained by other researchers, who achieved 49% reduction of CST after AD process of disintegrated sludge, compared to control sample [47]. Whereas, other scientists reported a 7-fold decrease of CST value (from 2000 s to 267 s) in the examined sludge, in compare to reference sample [48].

**Figure 5:**
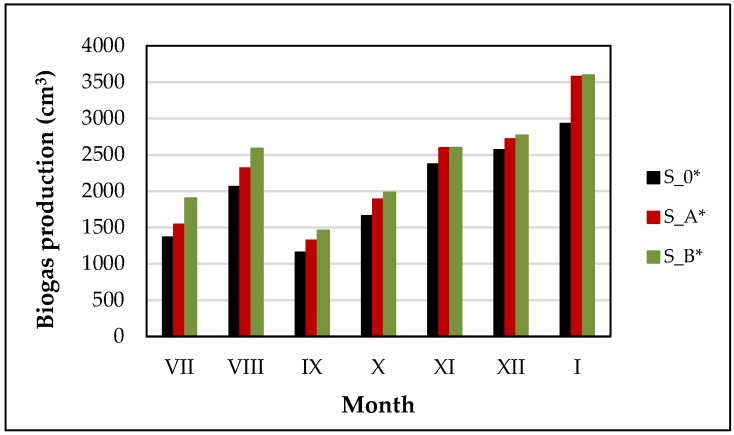
Biogas production after anaerobic digestion (AD) process.

The amount of total biogas production observed after the process of sludge anaerobic digestion is presented in Figure 5. Obtained results confirmed the positive effect of WAS ultrasonic pretreatment on the increase of biogas production. The higher volume of evolved biogas was obtained for a mixture containing disintegrated sludge, i.e., 13.0% (S_A*) and 19.7% (S_B*), in compare to control sample. The amount of biogas production did not undergoing a large variability during the experiment conducting, regardless of the technical conditions of WAS pretreatment, before AD process. The highest volume of evolved biogas was obtained from the mixture containing sludge after pretreatment in the ultrasonic washer (2411 cm^3^). The above observations were in a good agreement with the results obtained by other researchers. For example, it was stated that ultrasonic pretreatment of WAS at 20 kHz and P = 200 W, increased biogas production by 6.3% [40]. Whereas, other scientists achieved of 8.6% to 31.4% improvement of biogas production, conducting ultrasonic disintegration, at specific energy, in the range of: 15,000 to 35,000 kJ·kg^−1^ TS [10].

This study revealed, that there were no significant differences between the qualities of biogas originating from the mixtures containing untreated and disintegrated WAS. The concentrations of CH_4_, CO_2_ and H_2_S ranged from: 65.3 to 67.9% and 25.6 to 25.9%, as well as <1.0 ppm, respectively (Table 5). Similar composition of evolved biogas was indicated by other researchers, i.e., CH_4_ from 50 to 70%; CO_2_ from 25 to 30% and H_2_S, H_2_, N_2_ < 1% [49,50].

### 3.3. Statistical Analysis

In order to present the effects of ultrasonic disintegration as a function of WAS characteristics and technical conditions of the conducted process, a comprehensive analysis was carried out. In order to determine if there are any significant differences between examined variables, expressed as the changes in the characteristics of WAS before and after ultrasonic disintegration the *T*-Test was used. The strength of the relationship between periodical changes in the WAS characteristics (parameters) and obtained effects, was expressed as Pearson’s correlation coefficient (r). Moreover, to indicate the differences between effects obtained in various technical conditions (disintegrators) of process conducting, the one-way analysis of variance (ANOVA) was carried out.

#### 3.3.1. Analysis of Direct Effects

The results of *T*-Test confirmed existence of significant differences in the characteristics of WAS before and after ultrasonic pretreatment (*p* < 0.05). The only exceptions were: pH for samples disintegrated in WK-2010, as well as proteins for ultrasonic washer (*p* > 0.05) (Table A1).

The results of Pearson’s correlation are shown in Table A2 (WK-2010) and Table A3 (ultrasonic washer). A detailed analysis of correlation matrix revealed the existence of different relationships between the characteristics of sludge supernatant before and after WAS pretreatment. Moreover, apart from the statistically significant correlations, the ones for which “r” was higher than 0.65, were also taken into consideration. Taking into account the number of correlations between analyzed variables, it can be stated that the most important characteristics of WAS, which affects the magnitude of the direct effects, are: TS, VS, ΔT > EPS > SCOD > CST > N_TOT_, P_TOT_ > pH. Moreover, data analysis also indicated that, the increase of sludge temperature during ultrasonic pretreatment of WAS exerted a significant impact on its final characteristics.

#### 3.3.2. Analysis of Technological Effects

The *T*-Test results showed a significant differences in the characteristics of mixtures consisting of inoculum and pretreated WAS, prior and after anaerobic digestion (*p* < 0.05). The only exception was: CST for samples containing WAS after pretreatment in the ultrasonic washer (*p* > 0.05) (Table A4).

Furthermore, the results of Pearson’s correlation revealed statistically significant differences between the characteristics of mixtures prior and after completion of anaerobic digestion (Table A5 and Table A6). The above observations occurred regardless of the technical conditions of ultrasonic disintegration. In conclusion, it was found that the most important sludge parameters, which affect the magnitude of the technological effects, are: TS, VS > CST > N_TOT_, P_TOT_ > pH. The sequence of above parameters was determined based on the number of correlations between analyzed variables. Moreover, taking into account VS reduction and increase of biogas production, it can be said that increase of sludge temperature during ultrasonic disintegration most likely causes the difference in its biodegradation.

#### 3.3.3. Analysis of Technological Conditions of Process Conducting

The analysis of variance (ANOVA) showed that in the case of direct effects, there is a basis for rejecting the null hypothesis (H0) on the about the absence of statistically significant differences between the variables in considered groups (except pH, proteins and carbohydrates) (Table A7). It means, that above mentioned effects differ depending on the type of experimental device used in the process (*p* < 0.05). Whereas, the results of statistical analysis in accordance to technological effects indicated existence of significant differences between analyzed variables, but only in the case of changes in the CST values (Table A8). It means that technological effects are dependent on the characteristics of sludge subjected to ultrasonic disintegration, but only indirectly. However, in contradiction to results of the analysis of variance, the values of disintegration indicators indicated that there were a differences in the magnitude of technological effects depending on the type of experimental device used in the process of WAS ultrasonic disintegration.

In conclusion, according to the results of statistical analysis and values of applied indicators, it could be inferred that the most promising conditions for ultrasonic pretreatment of WAS (conducting at constant energy input), are: low power, long sonication time, large surface area of the emitter.

## 4. Conclusions

This article presents the results of a comprehensive analysis of direct and technological effects of disintegration as a function of periodical changes in the characteristics of WAS, as well as technical conditions of the conducted process. In this purpose selected parameters were considered, i.e., pH value, SCOD, N_TOT_, P_TOT_, proteins and carbohydrates concentrations, CST value, floc disruption, TS and VS content, before and after sludge ultrasonic disintegration or anaerobic digestion. To evaluation of direct and technological effects the commonly applied (DD_COD_) and author’s (IDi, IDd) indicators were used.

As a result of ultrasonic pretreatment, in the sludge or supernatant the increase of all examined parameters, except proteins and carbohydrates, were observed. Moreover, it was also showed that after completion of sludge anaerobic digestion, the increase of pH, N_TOT_ and P_TOT_ concentration, sludge dewaterability, as well as the reduction of TS and VS content and increases of biogas production were observed. Furthermore, the conducted experiment revealed that ultrasonic disintegration reduces the content of biogenic substances in the sludge supernatant after its anaerobic digestion. This information is positive, especially with respect to necessity of recycling the leachate, generated after dehydration of sludge undergone anaerobic digestion into the technological line.

The result of *T*-Test showed that there were significant differences between the characteristics of untreated and disintegrated WAS, regardless of the technological conditions of process conducting. The above observations were related both to direct and technological effects. Whereas, Pearson’s correlation confirmed, that changes in the characteristics of untreated WAS influencing effects of ultrasonic pretreatment. It was indicated that, the most important parameters, which affected the magnitude of the direct and technological effects, were: TS, VS, ΔT > EPS > SCOD > CST > N_TOT_, P_TOT_ > pH and TS, VS > CST > N_TOT_, P_TOT_ > pH, respectively. Furthermore, the analysis of variance showed, that the most significant differences between the effects obtained in various experimental devices were observed for: SCOD, N_TOT_, P_TOT_, CST, ΔT (direct effects) and CST (technological effects). It was found, that the most favorable effects of sludge ultrasonic pretreatment can be obtained conducting the process in the device characterized with low power, long sonication time and large surface area of the emitter. The results obtained in this study also confirmed the significant impact of the increase of sludge temperature during ultrasonic disintegration on the obtained effects.

## Figures and Tables

**Table 1:** The operational parameters of the Wastewater Treatment Plant.

Parameter ^1^	Unit	Value
Population equivalent (PE)	-	155,009
Average flow (Q)	m^3^·d^–1^	31,913
Hydraulic retention time (HRT) of sludge in anaerobic digester	d	33

^1^ Data for the year 2017 obtained from the Central WWTP in Gliwice.

**Table 2:** The list of methods used in this study.

Parameter	Methods/Devices	Reference	Type of Effects
pH	Electrometric method; Multi HQ40D (Hach Lange)	PN-EN 12176:2004 [27]	Direct; Technological
TS	Weight method (at 105 °C)	PN–EN 12880:2004 [28]	Technological;
VS	Weight method (at 550 °C)	PN–EN 12879:2004 [29]	Technological
SCOD_0_; SCOD_UD_	Potassium dichromate method; measurement tests LCI 400, LCK 014 (Hach Lange); UV–VIS DR 5000	ISO 15705:2002 [30]	Direct
N_TOT_	Potassium oxidation method measurement tests LCK 238 (Hach Lange); UV–VIS DR 5000	ISO 11905-1:1997 [31]	Direct, Technological
P_TOT_	Ammonium molybdate method; measurement tests LCK 350 (Hach Lange); UV–VIS DR 5000	ISO 6878:2004 [32]	Direct, Technological
Proteins	Folina—Ciocalteau reagent; UV–VIS DR 5000	Lowry et al., 1951 [33]	Direct
Carbohydrates	Phenol-Sulphuric acid reaction method; UV–VIS DR 5000	Dubois et al., 1956 [34]	Direct
CST	Quantity measurement; Capillary suction timer (Envolab)	PN–EN 14701–1:2007 [35]	Direct, Technological
ΔT	Quantity measurement; Digi—Sense (Cole—Parmer)	-	Direct
Flocs disruption	Optical microscopy; Optical microscope (MOTIC BA400)	-	Direct
Biogas production	Quantitative measurement; MULTITEC 540 gauge (Sewerin)	-	Technological
Biogas composition	Qualitative measurement; CH_4_ and CO_2_ (% vol.), H_2_S (ppm); MULTITEC 540 gauge (Sewerin)	-	-

TS—total solids; VS—volatile solids; SCOD_0_—SCOD of the supernatant of the original sludge; SCOD_UD_—SCOD value of the supernatant of the disintegrated sludge; TN—total nitrogen; TP—total phosphorus; CST—capillary suction time; ΔT—temperature increase.

**Table 3:** Technical characteristics and operating conditions of the experimental ultrasonic devices.

Parameter	Symbol	Unit	WK-2010	Ultrasonic Washer
Power	P	W	650	90
Frequency	f	kHz	25	25
Number of emitters	-	-	1	1
Emitter surface area	A_E_	cm^2^	78.5	19.6
Emitter diameter	d_E_	cm	10	5
Emitter position	h_E_	cm	1 ^1^	built
Chamber dimensions	idc × dc	cm	14 × 7 ^2^	15 × 13.7 × 20 ^3^
Chamber volume	V_C_	mL	1000	2000

^1^ emitter position relative to the sludge mirror; ^2^ dimension of chamber for sludge UD (ultrasonic disintegration) in relation to WK-2010 (internal diameter, depth); ^3^ internal dimensions of the ultrasonic washer (front, side, depth).

**Table 4:** The characteristics of the WAS before and after ultrasonic pretreatment—direct effects (n = 7).

Parameter	Unit	WAS_NUD	WAS_A	WAS_B
		Mean; CV		
pH	-	7.0 (2.0%)	6.9 (2.2%)	6.8 (2.7%)
TS	g·L^−1^	41.1 (26.9%)	-	-
VS	g·L^−1^	29.8 (29.2%)	-	-
SCOD_0_ /SCOD_UD_	mg·L^−1^	66.8 (22.8%)	1061.7 (34.8%)	4573.4 (39.2%)
N_TOT_	mg·L^−1^	10.9 (16.3%)	139.9 (23.8%)	470.4 (37.5%)
P_TOT_^1^	mg·L^−1^	25.1 (31.4%)	135.6 (22.5%)	177.0 (18.7%)
Proteins	mg·L^−1^	888.9 (24.9%)	784.0 (35.1%)	927.5 (29.2%)
Carbohydrates	mg·L^−1^	598.4 (35.6%)	480.7 (47.5%)	507.0 (46.9%)
CST	s	9.0 (24.8%)	343.1 (70.0%)	1175.1 (10.2%)
ΔT	°C	-	14.0 (39.7%)	34.0 (29.5%)

WAS_NUD—WAS before pretreatment; WAS_A—WAS after pretreatment in the WK-2010; WAS_B—WAS after pretreatment in the ultrasonic washer; CV—coefficient of variation; TS—total solids; VS—volatile solids; SCOD_0_—SCOD of the supernatant of the original sludge; SCOD_UD_—SCOD value of the supernatant of the disintegrated sludge; TN—total nitrogen; TP—total phosphorus; CST—capillary suction time; ΔT—temperature increase.

**Table 5:** The characteristics of the sludge sample before and after anaerobic digestion—technological effects.

Parameter	Unit	Inoculum	S_0	S_0*	S_A	S_A*	S_B	S_B*
		Mean; CV (%)						
pH	-	7.4 ± (1.7%)	7.5 (1.4%)	7.5 (1.1%)	7.3 (1.7%)	7.6 (1.2%)	7.3 (1.6%)	7.6 (1.3%)
TS	g·L^−1^	26.2 (15.1%)	36.8 (19.8)	30.2 (21.3%)	37.5 (19.0%)	29.2 (20.6%)	38.7 (17.9%)	28.6 (19.4%)
VS	g·L^−1^	15.9 (14.3%)	25.2 (20.2%)	18.7.1 (21.6%)	26.7 (20.5%)	18.2 (21.8%)	27.4 (18.8%)	17.7 (20.3%)
N_TOT_	mg·L^−1^	728.1 (11.3%)	210.6 (7.1%)	925.6 (18.5%)	277.0 (7.5%)	951.8 (19.4)	436.6 (21.9%)	1064.6 (16.8%)
P_TOT_	mg·L^−1^	116 (44.5%)	68.7 (52.6)	288.6 (54.7%)	130.4 (37%)	270.1 (60.3%)	145.8 (35.7%)	258.8 (64.1%)
CST	s	186.4 (26.6%)	42.6 (30.6%)	78.6 (63.8%)	352.4 (55.9%)	215.3 (43.8%)	1068.3 (10.7%)	342.7 (42.2%)
Total biogas production	cm^3^	-	-	2014.0 (32.4%)	-	2276.0 (34.0%)	-	2411.0 (29.0%)
CH_4_	%vol.	-	-	65.6 (5.7%)	-	66.0 (5.1%)	-	67.9 (4.6%)
CO_2_	%vol.	-	-	25.9 (16.6%)	-	25.6 (15.8%)	-	25.9 (12.1%)
H_2_S	ppm	-	-	<1.0	-	<1.0	-	<1.0

S_0—sample before AD containing inoculum and original sludge; S_0*—sample after AD containing inoculum and original sludge; S_A—sample before AD containing inoculum and WAS_A; S_A*—sample after AD containing inoculum and WAS_A; S_B—sample before AD containing inoculum and WAS_B; S_B*—sample after AD containing inoculum and WAS_B; TS—total solids; VS—volatile solids; N_TOT_—total nitrogen; P_TOT_—total phosphorus; CST—capillary suction time; methane ^(^CH_4_), carbon dioxide (CO_2_) and hydrogen sulfide (H_2_S) content in evolved biogas; vol.—volume.

## Appendix A

**Table A1:** The results of *T*-Test—direct effects.

Variables	WAS_A	WAS_B
**pH**	0.149546	**0.021015 ***
**SCOD**	**0.000456 ***	**0.000558 ***
**N_TOT_**	**0.000060 ***	**0.000463 ***
**P_TOT_**	**0.000037 ***	**0.000010 ***
**Proteins**	**0.018871 ***	0.478267
**Carbohydrates**	**0.000543 ***	**0.000457 ***
**CST**	**0.010617 ***	**0.000000 ***
**T**	**0.000540 ***	**0.000000 ***

* bold—significant, *p* < 0.05; SCOD—soluble chemical oxygen demand; N_TOT_—total nitrogen; P_TOT_—total phosphorus; CST—capillary suction time; T—temperature.

**Table A2:** Pearson’s correlation coefficients for the direct effects of WAS ultrasonic disintegration WK-2010).

Variables	pH	SCOD	N_TOT_	P_TOT_	Proteins	Carbohydrates	CST
**pH**	0.00	−0.37	−0.17	−0.35	−0.30	−0.05	−0.01
**SCOD**	−0.32	−0.75 **	−0.56	0.19	0.42	0.68 **	−0.71 **
**N_TOT_**	0.29	−0.72 **	−0.61	−0.10	0.37	**0.85 ***	**−0.87 ***
**P_TOT_**	−0.51	−0.20	−0.74	0.54	0.70	0.46	−0.39
**Proteins**	−0.68 **	−0.35	−0.27	**0.92 ***	**0.96 ***	0.57	−0.48
**Carbohydrates**	−0.21	**−0.88 ***	−0.43	0.45	**0.79 ***	**0.98 ***	**−0.97 ***
**CST**	−0.44	−0.65 **	−0.60	0.47	0.66 **	0.75 **	−0.72 **
**TS**	−0.31	**−0.85 ***	−0.54	0.37	0.70 **	**0.90 ***	**−0.94 ***
**VS**	−0.43	**−0.83 ***	−0.52	0.51	**0.80 ***	**0.90 ***	**−0.92 ***
**ΔT**	0.47	**0.84 ***	0.53	−0.52	**−0.80 ***	**−0.87 ***	**0.88 ***

* bold—significant correlations, *p* < 0.05; ** non-significant correlations taken into account in statistical analysis (r ≥ 0.65); SCOD—soluble chemical oxygen demand; N_TOT_—total nitrogen; P_TOT_—total phosphorus; CST—capillary suction time; ΔT—temperature increase.

**Table A3:** Pearson’s correlation coefficients for the direct effects of WAS ultrasonic disintegration (ultrasonic washer).

Variables	pH	SCOD	N_TOT_	P_TOT_	Proteins	Carbohydrates	CST
**pH**	0.21	0.44	0.60	−0.16	−0.36	−0.07	−0.24
**SCOD**	−0.28	0.04	0.06	0.25	0.33	0.65 **	**−0.79 ***
**N_TOT_**	0.29	0.22	0.02	−0.01	0.53	**0.84 ***	−0.58
**P_TOT_**	−0.42	−0.55	−0.51	0.55	0.51	0.44	−0.34
**Proteins**	−0.66 **	**−0.92 ***	**−0.95 ***	**0.95 ***	**0.87 ***	0.58	−0.58
**Carbohydrates**	−0.21	−0.31	−0.47	0.56	**0.88 ***	**0.98 ***	**−0.91 ***
**CST**	−0.48	−0.28	−0.31	0.44	0.57	0.73 **	−0.74 **
**TS**	−0.28	−0.17	−0.26	0.46	0.67	**0.88 ***	**−0.91 ***
**VS**	−0.40	−0.32	−0.40	0.60	0.75 **	**0.89 ***	**−0.94 ***
**ΔT**	0.59	0.43	0.50	−0.71 **	**−0.77 ***	**−0.76 ***	**0.94 ***

* bold—significant correlations, *p* < 0.05; ** non-significant correlations taken into account in statistical analysis (r ≥ 0.65); SCOD—soluble chemical oxygen demand; N_TOT_—total nitrogen; P_TOT_—total phosphorus; CST—capillary suction time; ΔT—temperature increase.

**Table A4:** The results of *T*-Test—technological effects.

Variables	WAS_A	WAS_B
**pH**	**0.019198 ***	**0.000932 ***
**TS**	**0.000013 ***	**0.000003 ***
**VS**	**0.000009 ***	**0.000005 ***
**N_TOT_**	**0.000113 ***	**0.000463 ***
**P_TOT_**	**0.023667 ***	**0.047379 ***
**CST**	0.191863	**0.000351 ***

* bold—significant, *p* < 0.05; TS—total solids; VS—volatile solids; N_TOT_—total nitrogen; P_TOT_—total phosphorus; CST—capillary suction time.

**Table A5:** Pearson’s correlation coefficients for the technological effects of WAS ultrasonic disintegration (inoculum + WAS_A).

Variables	pH	TS	VS	N_TOT_	P_TOT_	CST	^3^ Biogas
**pH**	−0.44	0.03	−0.19	−0.26	−0.65 **	−0.18	−0.42
**TS**	−0.53	**0.98 ***	**0.99 ***	**0.92 ***	0.57	0.74 **	**0.85 ***
**VS**	−0.34	**0.92 ***	**0.99 ***	**0.97 ***	0.73 **	**0.81 ***	**0.94 ***
**N_TOT_**	0.20	−0.67 **	−0.75 **	−0.73 **	−0.74 **	−0.64	**−0.79 ***
**P_TOT_**	0.47	0.22	0.48	0.63	**0.88 ***	0.59	0.74 **
**CST**	0.61	**−0.91 ***	**−0.88 ***	−0.75 **	−0.37	−0.66 **	**−0.77 ***

* bold—significant correlations, *p* < 0.05; ** non−significant correlations taken into account in statistical analysis (r ≥ 0.65); Biogas—total biogas production; TS—total solids; VS—volatile solids; N_TOT_—total nitrogen; P_TOT_—total phosphorus; CST—capillary suction time.

**Table A6:** Pearson’s correlation coefficients for the technological effects of WAS ultrasonic disintegration (inoculum + WAS_B).

Variables	pH	TS	VS	N_TOT_	P_TOT_	CST	Biogas
**pH**	−0.02	−0.11	−0.32	−0.19	−0.75 **	−0.31	−0.43
**TS**	−0.64	**0.99 ***	**0.93 ***	**0.88 ***	0.29	**0.78 ***	**0.76 ***
**VS**	−0.63	**0.97 ***	**0.99 ***	**0.92 ***	0.50	**0.87 ***	**0.89 ***
**N_TOT_**	−0.16	−0.26	−0.51	−0.49	**−0.94 ***	−0.62	**−0.76 ***
**P_TOT_**	0.17	0.15	0.41	0.31	**0.91 ***	0.57	0.63
**CST**	0.28	**−0.81 ***	**−0.92 ***	**−0.94 ***	−0.72 **	**−0.88 ***	**−0.93 ***

* bold—significant correlations, *p* < 0.05; ** non−significant correlations taken into account in statistical analysis (r ≥ 0.65); Biogas—total biogas production; TS—total solids; VS—volatile solids; N_TOT_—total nitrogen; P_TOT_—total phosphorus; CST—capillary suction time.

**Table A7:** The results of ANOVA Test—direct effects.

Variable	Unit	*p* Value
**pH**	-	0.233545
**SCOD**	mg·L^−1^	**0.000273 ***
**N_TOT_**	mg·L^−1^	**0.000387 ***
**P_TOT_**	mg·L^−1^	**0.031615 ***
**Proteins**	mg·L^−1^	0.344718
**Carbohydrates**	mg·L^−1^	0.729555
**CST**	s	0.000003
**ΔT**	°C	**0.000001 ***

* bold—significant correlations, *p* < 0.05; SCOD—soluble chemical oxygen demand; N_TOT_—total nitrogen; P_TOT_—total phosphorus; CST—capillary suction time; ΔT—temperature increase.

**Table A8:** The results of ANOVA Test—technological effects.

Variable	Unit	*p* Value
**pH**	-	0.082577
**TS**	g·L^−1^	0.884421
**VS**	g·L^−1^	0.792592
**N_TOT_**	mg·L^−1^	0.324162
**P_TOT_**	mg·L^−1^	0.942013
**CST**	s	**0.000801 ***
**Biogas production**	cm^3^	0.577505

* bold—significant correlations, *p* < 0.05, TS—total solids; VS—volatile solids; N_TOT_—total nitrogen; P_TOT_—total phosphorus; CST—capillary suction time.
